# Structural plasticity of pyramidal cell neurons measured after FLASH and conventional dose-rate irradiation

**DOI:** 10.1007/s00429-025-02902-y

**Published:** 2025-03-01

**Authors:** Dara L. Dickstein, Richard Zhang, Ning Ru, Marie-Catherine Vozenin, Bayley C. Perry, Juan Wang, Janet E. Baulch, Munjal M. Acharya, Charles L. Limoli

**Affiliations:** 1 Department of Pathology, Uniformed Services University of Health Sciences, Bethesda, MD 20814 USA; 2 The Henry M. Jackson Foundation for the Advancement of Military Health Inc, Bethesda, MD USA; 3 Department of Radiation Oncology, University of California, Irvine School of Medicine, Irvine, CA USA; 4 Secteur Radio-Oncologie et Radiobiologie, Hôpitaux Universitaires de Genève, Geneva, Switzerland; 5 Department of Anatomy and Neurobiology, University of California, Irvine School of Medicine, Irvine, CA USA

**Keywords:** (4–6): FLASH, Radiotherapy, Cranial irradiation, Neuron, Structural plasticity

## Abstract

Evidence shows that ultra-high dose-rate FLASH-radiotherapy (FLASH-RT) provides relative protection against normal tissue complications and functional decrements in the irradiated brain. Past work has shown that radiation-induced cognitive impairment, neuroinflammation and reduced structural complexity ofgranule cell neurons were not observed to the same extent after FLASH-RT (> MGy/s) compared to conventional dose-rate (CONV, 0.1 Gy/s) delivery. In this study, we explored the sensitivity of hippocampal CA1 and medial prefrontal cortex (mPFC) pyramidal neurons to cranial irradiation and dose-rate modulation using electron and confocal microscopy. Neuron ultrastructural analyses by electron microscopy after 10 Gy FLASH- or CONV-RT exposures indicated that irradiation had little impact on dendritic complexity and synapse density in the CA1, but did increase the length and head diameter of smaller non-perforated synapses. Similarly, irradiation caused no change in mPFC prelimbic/infralimbic axospinous synapse density, but reductions in non-perforated synapse diameters. While irradiation resulted in thinner myelin sheaths compared to controls, none of these metrics were dose-rate sensitive. Analysis of fluorescently labeled CA1 neurons revealed no radiation-induced or dose-rate-dependent changes in overall dendritic complexity or spine density, in contrast to our past analysis of granule cell neurons. Super-resolution confocal microscopy following a clinical dosing paradigm (3 × 10 Gy) showed significant reductions in excitatory vesicular glutamate transporter 1 and inhibitory vesicular GABA transporter puncta density within the CA1 that were largely dose-rate independent. Collectively, these data reveal that, compared to granule cell neurons, CA1 and mPFC neurons are relatively more radioresistant irrespective of radiation dose-rate.

**Supplementary Information:**

The online version contains supplementary material available at 10.1007/s00429-025-02902-y.

## Introduction

The complexity of the dendritic tree and the interconnections between dendritic spines define a myriad of synaptic capabilities that mediate neurotransmission. In the adult rodent brain, age, disease and cancer treatment-associated changes can compromise the integrity of neuronal structure and adversely impact cognition (Selkoe 2002; Dickstein et al. 2007, 2010, 2013; Seigers and Fardell 2011; Vogel-Ciernia et al. 2013; Christie et al. 2012; Parihar and Limoli 2013; Baulch et al. 2016). Among these treatments, cranial radiotherapy and systemic chemotherapy represent frontline treatments for forestalling the growth of brain malignancies and disseminated, oligometastatic disease (Evenden 2013; Wefel and Schagen 2012; Makale et al. 2017). In rodent models, each of these treatments elicits qualitatively similar adverse neurocognitive sequelae invariably associated with elevations in neuroinflammation along with macroscopic changes in dendritic complexity, microscopic changes in synapse morphology and changes in neuronal structural elements including dendritic spines, axonal myelination and synaptic bouton (Chakraborti et al. 2012; Acharya et al. 2015a, b; Parihar et al. 2015; Dickstein et al. 2018; Allen et al. 2022). The majority of past work has focused on granule cell neurons in the hippocampal dentate that exhibit exquisite sensitivity to photon, electron and proton irradiation delivered at conventional dose-rates (CONV, 2 Gy/min) (Parihar and Limoli 2013; Parihar et al. 2015, 2016; Baulch et al. 2016; Simmons et al. 2019; Montay-Gruel et al. 2019). Studies implementing a wide range of radiation paradigms have shown that doses ranging from 0.3 to 10 Gy elicit marked and persistent reductions in dendritic arborization, immature dendritic spine density, spinogenesis and myelination at protracted 1 to 9 month post-irradiation times (Parihar and Limoli 2013; Parihar et al. 2015; Baulch et al. 2016; Dickstein et al. 2018; Montay-Gruel et al. 2019; Alaghband et al. 2023b). Interestingly, when granule cell neurons were analyzed at similar times following exposure to electron FLASH-radiotherapy (FLASH), these same reductions in dendritic complexity and spine density where not found at doses as high as 10 Gy (Montay-Gruel et al. 2019).

While granule cell neurons and the cellular constituents within the neurogenic niches of the mammalian brain have been studied extensively over decades (van Praag et al. 2002) (Tofilon and Fike 2000) (Mizumatsu et al. 2003), differences in the radiosensitivity and structural plasticity of relatively more mature neuronal populations have not. Further, whether mature populations of neurons exhibit sensitivity to changes in dose-rate that characterize CONV and FLASH irradiation modalities remains equally unexplored. Post-mitotic neurons comprising the majority of the brain have been studied in aging and neurodegenerative conditions using combinations of fluorescently-labeled mouse models (Parihar and Limoli 2013), dye loading (Price et al. 2014) and electron microscopic (EM) techniques (Bloss et al. 2013). These approaches have been used by our group to characterize radiation-induced changes in the hippocampal dentate (Parihar and Limoli 2013; Price et al. 2014), but not for principal cells in other regions of the brain or after exposure to CONV and FLASH electron radiations. Thus, the focus of the current study was twofold, **(1)** to address whether relatively mature and arbored subsets of neurons in the pyramidal layer of the CA1 and prelimbic/infralimbic region of the medial prefrontal cortex (PFC) exhibited similar sensitivities to radiation exposure and **(2)** whether they were responsive to dose-rate modulation as observed for granule cell neurons (Montay-Gruel et al. 2019). Ultrastructural analyses of both neuronal populations by EM showed these neurons to be largely resistant to radiation-induced change, findings that were corroborated by analysis of fluorescently labeled neurons in the CA1 by confocal microscopy. Furthermore, while analyses of excitatory vesicular glutamate transporter 1 and inhibitory vesicular GABA transporter (i.e. glutamatergic/GABAergic VGLUT/VGAT) puncta in the CA1 revealed dose-dependent reductions in synapse density, they were not found to depend on dose-rate. Here we report on the marked radioresistance of pyramidal neurons to structural alterations following clinically relevant radiation doses delivered at either CONV or FLASH dose-rates.

## Materials and methods

### Animals and irradiations

Animal experiments were approved by the Swiss (Vaud state approval: VD2920, 3241 and 3603) and University of California, Irvine (Institutional Animal Care and Use Committee: AUP 23–080) ethics committees for animal experimentation and follow ARRIVE guidelines and address the 10 essential criteria described therein.

Prior work with female mice revealed marked radiation-induced changes in granule cell neurons that were dose rate dependent (Montay-Gruel et al. 2017). Here, initial studies focused on female mice, to compare possible differences in the structural sensitivity of CA1 neurons to dose and dose rate dependent changes by a dye loading technique. Female C57Bl/6 mice were purchased from Charles River Laboratories at 8 weeks of age. Tumor-free female transgenic mice (Tg(Thy1-eGFP) MJrsJ, stock no. 007788; The Jackson Laboratory) were bred at the University of California, Irvine animal facility. Mice received whole-brain irradiations (WBI) using the Oriatron eRT6 (PMB-Alcen) at 10 Gy, under isoflurane anesthesia where the mouse head was positioned behind and in contact with the aperture of the 1.7-cm-diameter graphite applicator to irradiate the brain at either CONV dose-rate (0.09 Gy/second) or ultra-high dose-rate FLASH delivered in a single 1.8 µs pulse (5.6 × 10^6^ Gy/second) thus irradiating the whole encephalon region, while limiting the dose to the eyes, the mouth, and the rest of the body (Montay-Gruel et al. 2017). The brains of these mice were prepared for EM and confocal microscopy 6 months after irradiation.

Prior studies using a more clinically relevant fractionated regimen, have found certain differences between the sexes (Alaghband et al. 2023a; Allen et al. 2022). To explore possible sex differences with past results, a separate cohort of male and female C57Bl/6 mice (*n* = 4/treatment/sex) were also included, and were purchased from Charles River Laboratories (France, strain code 632) and allowed to acclimate. For this cohort, mice were 10 weeks of age at the time of exposure and irradiation procedures were identical to those described above, except that these mice received three doses of 10 Gy (Mon, Wed, Fri) to a total dose of 30 Gy. These animals were used for the synaptic puncta analyses described below.

### Neuronal reconstruction

For 3-dimensions neuronal reconstructions, intracellular injections of individual CA1 hippocampal neurons were performed as previously described (Krishnan et al. 2021; Dickstein et al. 2018). Briefly, sections were incubated in 4’,6-diamidino-2-phenylindole (DAPI; Vector Labs) to reveal the cytoarchitectural features of the pyramidal layer of the CA1. The sections were mounted on nitrocellulose paper, immersed in ice-cold 0.1 M PBS and pyramidal neurons were subjected to an intracellular iontophoretic injection of 5% Lucifer Yellow (Invitrogen) under a direct current of 3–8 nA until dye had completely filled distal processes (Krishnan et al. 2021; Price et al. 2014; Steele et al. 2014). Five to 10 neurons were injected per slice and placed far enough apart to avoid overlapping of their dendritic trees. Brain sections were then mounted on gelatin-coated glass slides and cover slipped in Fluoromount G slide-mounting media (Southern Biotech).

Intact filled neurons were manually traced and reconstructed in with a 63×/1.4 N.A., Plan-Apochromat oil immersion objective on a Zeiss Axio Imager Vario microscope equipped with a motorized stage, video camera system, and Neurolucida morphometry software (MBF Bioscience). To be included in the analysis, a loaded neuron had to satisfy the following criteria: **(1)** reside within the pyramidal layer of the CA1 as defined by cytoarchitectural characteristics; **(2)** demonstrate complete filling of dendritic tree, as evidenced by well-defined endings; and **(3)** demonstrate intact tertiary branches, with the exception of branches that extended beyond 50 μm in radial distance from the cell soma (Krishnan et al. 2021; Price et al. 2014; Steele et al. 2014). Using NeuroExplorer software (MBF Bioscience) total dendritic length, number of intersections, and the amount of dendritic material per radial distance from the soma, in 30-µm increments (Sholl 1953), were analyzed in order to assess morphological cellular diversity and potential differences between the animal groups. A total of 50 cells were reconstructed for controls (~ 8 cells per animal) and a total of 49 cells were reconstructed for the irradiated mice (~ 8 cells per animal).

### Ultrastructural analysis of synapses and myelinated axons with electron microscopy

Coronal sections encompassing the CA1 region of the hippocampus and the medial PFC were prepared for EM as reported previously (Krishnan et al. 2021; Price et al. 2014; Steele et al. 2014; Alaghband et al. 2023b; Dickstein et al. 2018). Brain slices (250 μm-thick) were cryoprotected in graded phosphate buffer/glycerol washes at 4 °C, manually microdissected into 1 mm blocks, rapidly freeze-plunged into liquid propane cooled by liquid nitrogen (− 190 °C) in a universal cryofixation system KF80 (Reichert-Jung, Leica Microsystems, Wetzlar, Germany) and subsequently immersed in 1.5% uranyl acetate dissolved in anhydrous methanol at − 90 °C for 24 h in a cryosubstitution unit (Leica). Block temperatures were raised from − 90 to − 45 °C in steps of 4 °C per hour. Blocks were washed with anhydrous methanol, and infiltrated with Lowicryl resin (Electron Microscopy Sciences) at − 45 °C, polymerized by exposure to ultraviolet light (360 nm) for 48 h at − 45 °C followed by 24 h at 0 °C. Block faces were trimmed and ultrathin Sect. (90 nm) were cut with a diamond knife (Diatome) on an ultramicrotome (Reichert-Jung). Tissue grids were imaged on a JEOL JEM-1011 TEM (JEOL USA Inc, Peabody, MA) with an AMT XR50S-A camera (Advanced Microscopy. Techniques, Woburn, MA).

For synapse quantification, serial section micrographs were imaged at 15,000×. An unbiased stereological approach using the physical disector was performed to measure synapse density, as described in our previous work (Lazarczyk et al. 2023; Krishnan et al. 2021; Alaghband et al. 2023b; Dickstein et al. 2018) Nine sets of serial images across the same set of five consecutive ultrathin sections were taken for each animal and imported into Adobe Photoshop (version CC 2018 19.1.2, Adobe Systems, San Jose, CA). All axospinous synapses were identified within the first two and the last two images of each five-section serial set, and counted if they were contained in the reference image but not in the corresponding look-up image. To increase sampling efficiency, the reference image and look-up image were then reversed, thus each animal included in the current study contributed synapse density data from a total of 18 disector pairs. Axospinous synapse density was calculated as the total number of unique counted synapses from both images divided by the total volume of the disector (area × height of disector). The criteria for inclusion as an axospinous synapse included the presence of a presynaptic terminal and a distinct postsynaptic density (PSD) separated by a clear synaptic cleft. The same volume was sampled for each group. In addition to total synapse density, we also measured the densities of nonperforated and perforated synapses. Perforated synapses were defined by the presence of a discontinuity in the PSD. A single person, blinded to each of the treatment groups, performed all analyses.

Quantification of myelination was performed as previously described (Dickstein et al. 2018; Krishnan et al. 2021; Alaghband et al. 2023b). Briefly, to characterize the degree of myelination, the numbers of myelinated and unmyelinated axons were counted in 12 randomly selected, nonoverlapping fields of the hippocampal sulcus from each animal at 10,000×. Both the number of myelinated axons per square millimeter and the percent of total myelinated axons were calculated. An additional six randomly selected, nonoverlapping images were taken per animal at 15,000 × to evaluate myelin sheath thickness through g-ratio analysis. Four measurements were recorded for each myelinated axon: the longest axon diameter, the shortest axon diameter, the longest myelin width, and the shortest myelin width. To calculate the g-ratio, the average diameter for each axon was divided by the average axon diameter plus twice the average myelin width (Dupree et al. 2015; Murcia-Belmonte et al. 2016). Myelin regions that exhibited fixation artifacts or noncompaction were excluded from the analysis. A single person, blinded to the treatment groups, performed all analyses.

### Immunohistochemistry, confocal microscopy, and quantification

Hemizygous *Thy1-EGFP* mice expressing eGFP provided a fluorescent signal facilitating neuronal micromorphometric analysis as described previously (Parihar and Limoli 2013; Parihar et al. 2015). Briefly, 100 μm-thick hippocampal sections were cut for dendritic confocal imaging and analysis using a cryostat (Leica Microsystems). Three sections per animal were used to generate Z-stacks from four animals using a Nikon C2 confocal microscope. Images comprising each Z-stack (1,024 × 1,024 pixels) were acquired (100×) over the entire dendrite tree at 0.25-µm increments. Detailed dendritic tracing and spine classification was performed using the Imaris 10.1 software suite (Bitplane, Inc./Oxford Instruments). For spines to be included in our analyses, they required a minimal length of 0.3 μm and a maximal length of 1.8 μm. Parameters of neuronal structure that were identified and quantified through image reconstruction and deconvolution using the Imaris software suite included the cell body, dendritic and axonal length, branching and branch points, dendritic complexity, spines, and boutons. Spine classification was performed on Imaris using the filaments tool. Spine head and neck parameters were set as follows: Long-thin (neck length < 2 μm, mean head width < 0.35 μm), Mushroom (neck length < 1 μm, mean head width > 0.35 μm) and Stubby (spine length < 0.5 μm, mean head width > 0.35 μm).

Animals were prepared for immunohistochemistry as described previously (Allen et al. 2022; Alaghband et al. 2023a). Briefly, brains were sectioned coronally (30–35 μm thick) using a cryostat (Leica Microsystems) for super resolution microscopy and two 30 μm sections per animal containing the dorsal hippocampus were washed with PBS, before being permeabilized in a solution of 0.3% TX-100. Sections were then blocked in a solution of 10% NGS in PBS with 0.3% TX-100 and 4% BSA for 1 h and incubated overnight with VGLUT and VGAT primary antibodies (1:500, Synaptic Systems, Cat No. 135304, Cat No. 131003), in a solution of 0.3% TX-100, 10% NGS, and 1x PBS. Sections were then washed and incubated with secondary antibodies (1:1000 Invitrogen Anti-Guinea Pig AlexaFluor 488 A11073, 1:1000 Invitrogen Goat Anti-Rabbit AlexaFluor 555 A21428), before counterstaining with DAPI, and mounting. Sections were imaged at 63× on an Elyra 7 Super resolution microscope (Zeiss), focused on the apical CA1 region of the hippocampus. Images were captured at a resolution of 1280 × 1280 comprising of a Z-stack consisting of images taken at 273 nm intervals. Images were then processed using the Zeiss Zen Black Software’s SIM^2^ feature, to create super-resolution images of VGLUT and VGAT Puncta. Puncta were then quantified using Imaris 10.1 image analysis software via the Spots function. Spots larger than 180 nm were identified as puncta and counted. The number of puncta observed were averaged per animal, for a total of 8 animals per treatment.

### Statistics

For neuronal structure analyses, following confirmation of normal Gaussian distribution, one-way ANOVAs were used to assess significance between control and irradiated groups. A Tukey’s post hoc test was used to compare CONV-RT and FLASH-RT groups against the control group when overall group effects were found to be statistically significant. Two-way repeated measures were performed for Sholl analyses. For synapse density (total, perforated and non-perforated), following confirmation of normal Gaussian distribution, we performed one-way ANOVAs followed by Tukey’s multiple comparison when significance was achieved. The α level was set at 0.05 with values of *p* < 0.05 considered statistically significant. All data are reported as mean ± SEM. Statistical significance was calculated using GraphPad Prism 8 (San Diego, CA).

For the myelin analysis, to account for the nested data produced by g-ratio quantification, differences between treatment groups were evaluated using a linear mixed-effect model (LMM) regression analysis approach. LMMs were fit in R 4.1.2 using the lme4 (Bates et al. 2015) and lmerTest (Kuznetsova et al. 2017) packages, where outcome measures were analyzed against treatment fixed effects and a random effect for animal ID, representing the nested variation from multiple synapse or axon measurements per animal. Significant interaction effects were decomposed by comparison of estimated marginal means with the demeans package in R (Length 2022). Results were expressed as mean ± SEM and all analyses considered a value of *p* < 0.05 to be statistically significant.

## Results

### Radiation treatment affects synapse morphology but has no effect on synapse density or neuronal complexity

We assessed whether neurons from FLASH and CONV irradiated mice would display differences in dendritic length and complexity, synapse density and morphology compared to neurons from control mice. We found no significant difference in apical or basal dendritic length and neuronal complexity between all three treatment groups (Supplemental Fig. 1).

**Fig. 1:**
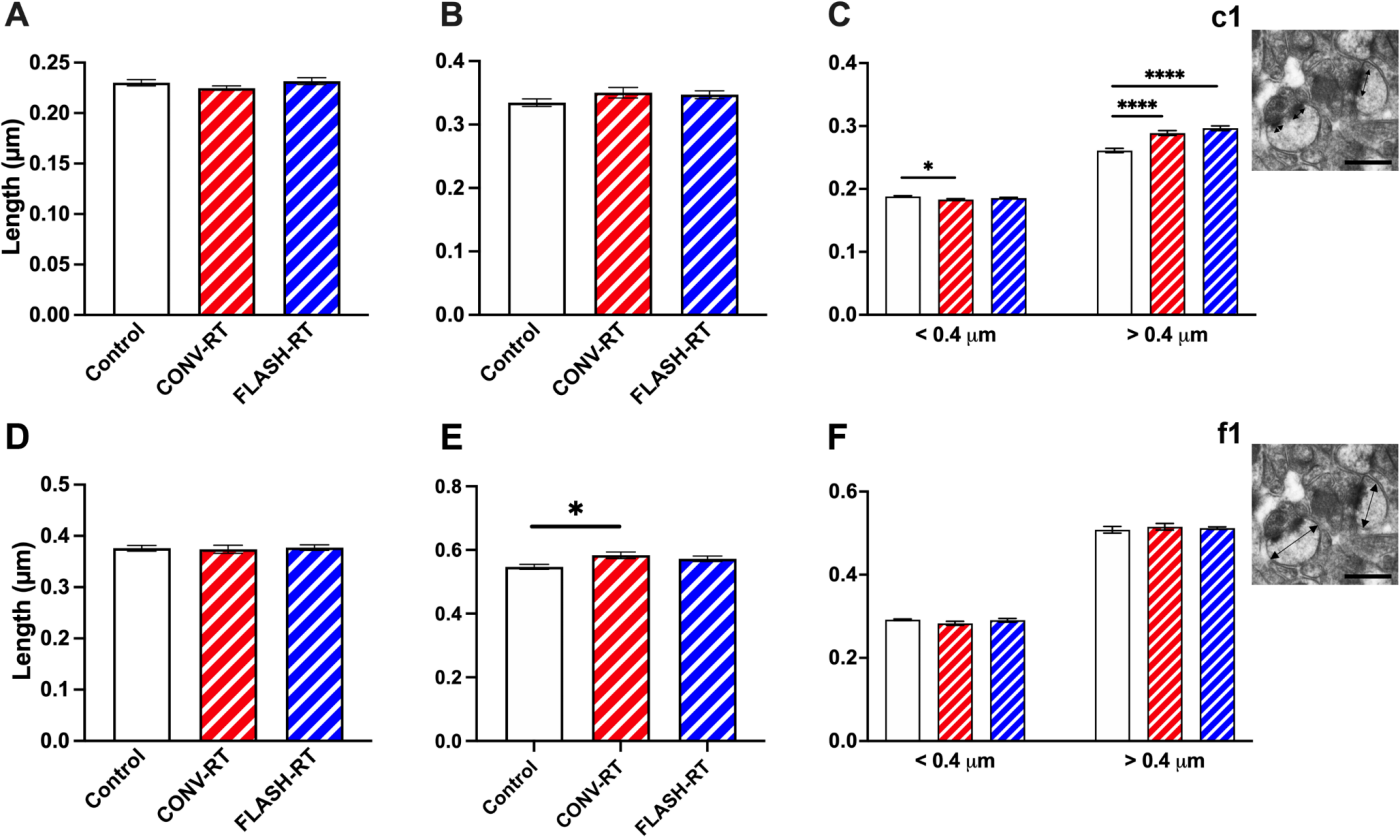
Radiation treatment affect PSD size and synapse diameter in the CA1 region of the hippocampus. Morphological analysis of synapses did not reveal significant differences in overall PSD length (**A**), or PSD length in perforated synapses (**B**), however, there were significant reductions in smaller non-perforated synapses (**C**). We did not observe any differences in spine head diameter in total synapses (**D**) however, when broken into perforated synapses (**E**) and non-perforated synapses (**F**) we found that perforated synapses in the CONV treated mice had larger head diameters than controls. Inset images depict PSD length (arrows in **c1**) and spine head diameter (arrows in **f1**). Data represents group mean ± SEM. *p < 0.05, ****p < 0.0001. Scale bars = 50 µM

To study the axospinous synapse density in the *stratum radiatum* (SR) region of the hippocampus, total synapse density as well as the density of perforated and non-perforated synapses were examined. Approximately 11,202 synapses were counted from all groups (747 spines per animal on average) across 9 serial EM sections using the dissector method (Table 1). We next examined the length and area of the PSD as well as the head diameter of individual spines. For PSD length, there was no difference between the groups (Fig. 1A). Measurement of spine head diameter also revealed no significant difference between control and CONV or FLASH mice (Fig. 1D). Our previous studies have classified mouse spines with head diameters < 0.4 μm as thin spines and > 0.4 μm as mushroom spines (Dickstein et al. 2018; Krishnan et al. 2021; Lazarczyk et al. 2016; Price et al. 2014; Steele et al. 2014). We applied this parameter to the PSD length and head diameter to non-perforated and perforated synapses. We found no difference in PSD length between groups in perforated synapses but did observe significant differences in non-perforated synapses (Fig. 1B, C respectively). In particular, in synapses < 0.4 mm CONV treated animals had smaller PSD than sham controls (F_(2, 3571)_ = 3.570, *p* = 0.028; Fig. 1C), while synapses > 0.4 mm both CONV and FLASH treated animals had larger PSDs compared to SHAM controls (F_(2, 1649)_ = 27.30, *p* < 0.0001; Fig. 1C). When we examined head diameter, CONV treated mice had significantly larger head diameters compared to controls in perforated synapses (F_(2, 471)_ = 4.246, *p* = 0.015; Fig. 1E). There was no difference in head diameter in non-perforated synapses (Fig. 1F).

**Table 1:** Summary of the numbers and quantitative morphological data of synapses and myelinated axons analyzed for each treatment group derived from EM

		Control		CONV	FLASH	
**CA1 Synapses**		Mean ± SEM		Mean ± SEM	Mean ± SEM	
Total synapse density		2.375 ± 0.202		2.839 ± 0.101	2.831 ± 0.153	
Perforated synapse density		0.413 ± 0.039		0.405 ± 0.056	0.410 ± 0.018	
Non-perforated synapse density		2.32 ± 0.169		2.434 ± 0.086	2.420 ± 0.146	
PSD length		0.230 ± 0.003		0.225 ± 0.002	0.232 ± 0.003	
PSD Area		0.037 ± 0.04		0.031 ± 0,001	0.033 ± 0.001	
Spine head diameter		0.376 ± 0,06		0.374 ± 0.008	0.377 ± 0.005	
**PFC Synapses**						
Total synapse density		1.117 ± 0.086		1.067 ± 0.050	1.017 ± 0.084	
Perforated synapse density		0.220 ± 0.021		0.171 ± 0.006	0.159 ± 0.026	
Non-perforated synapse density		0.896 ± 0.077		0.896 ± 0.050	0.856 ± 0.063	
PSD length (mm)		0.312 ± 0.012		0.279 ± 0.003	0.283 ± 0.008	
PSD Area (mm)		0.025 ± 0.001		0.022 ± 0.001	0.023 ± 0.001	
Spine head diameter (mm^2^)		0.523 ± 0.017		0.443 ± 0.005	0.459 ± 0.008	
**Myelin**						
Number myelinated axons		2392		1939	1906	
Number of axons for g-ratio		530		607	503	

**Fig. 2:**
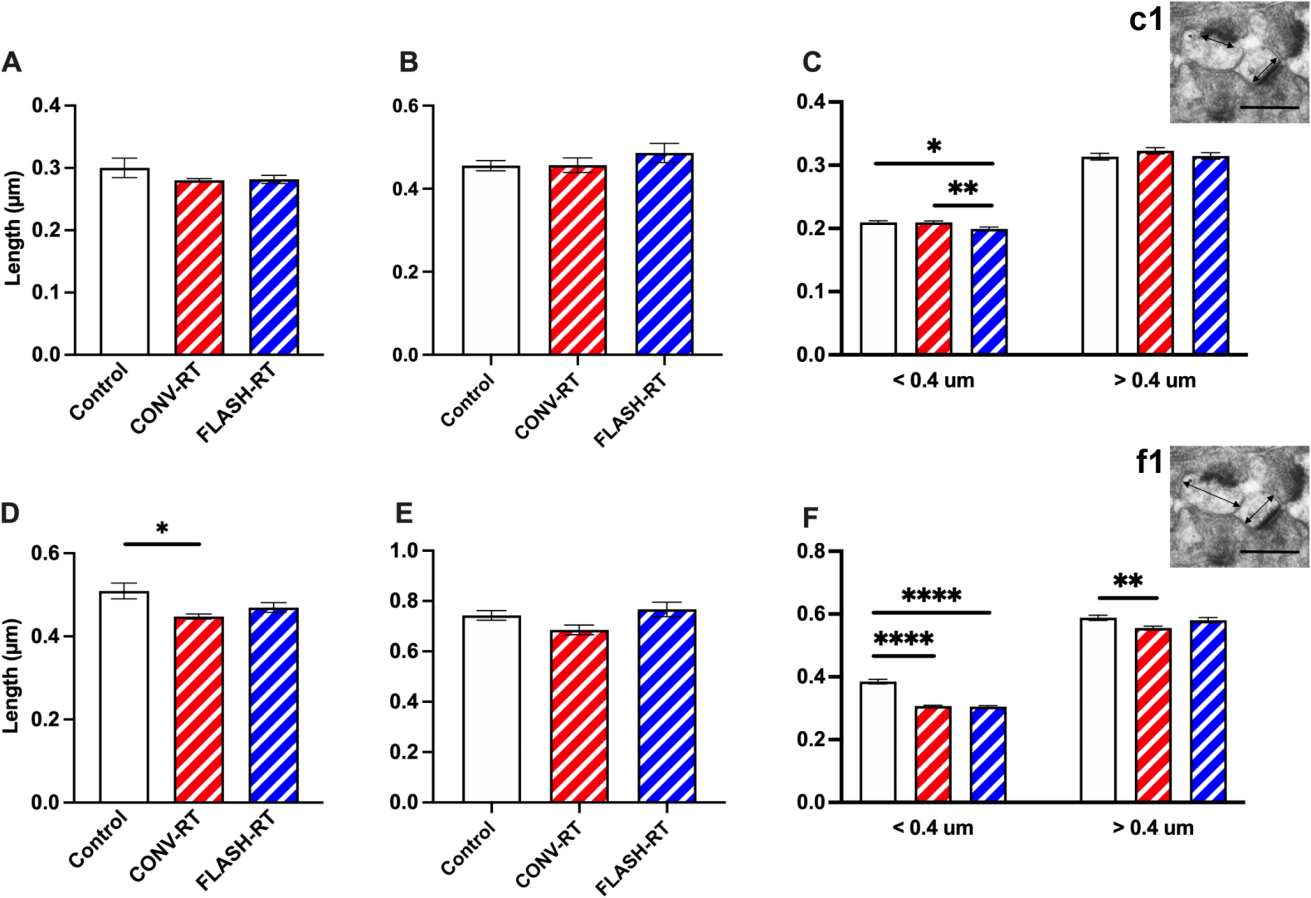
Radiation treatment affects PSD size and synapse diameter in the mPFC. Morphological analysis of synapses did not reveal significant differences in overall PSD length (**A**), or PSD length in perforated synapses (**B**), however, there was significant differences in smaller non-perforated synapses (**C**). We did see significant decrease in spine head diameter is total synapses (**D**) however, when broken into perforated synapses (**D**) and non-perforated synapses (**E**) we only saw differences in non-perforated synapses with irradiated animals having smaller head diameters than sham controls (**F**). Inset images depict PSD length in (**c1**) and head diameter in (**f1**). Data represents group means ± SEM. **p < 0.01, ****p < 0.0001. Scale bars = 50 µM

As was done for the hippocampus, we also examined the axospinous synapse density in the prelimbic/infralimbic region of the mPFC. Approximately 6502 unique synapses were studied from all groups (~ 507 synapses per animal *n* = 5 animals/group; see Table 1 for synapse measurements). Analysis of total perforated and non-perforated synapse density revealed no significant differences between control mice, CONV mice and FLASH mice (Supplemental Fig. 2). We next examined PSD length as well as the head diameter (HD) of the spine. We found no difference in overall length of the PSD in the irradiated mice compared to control mice (F_(2, 12)_ = 1.259, *p* = 0.3188, one-way ANOVA; Fig. 2A). Measurement of spine head diameter revealed a significant difference between groups (F_(2, 12)_ = 5.480, *p* = 0.020, one-way ANOVA; control vs. CONV: *p* = 0.017, Control vs. FLASH: *p* = 0.129, Bonferroni’s multiple comparison tests, Fig. 2D). When separating synapses by size as performed above, we found that there was no difference in perforated PSD length (F_(2, 253)_ = 0.8765, *p* = 0.4175, one-way ANOVA, Fig. 2B) but a significant difference in HD (F_(2, 253)_ = 3.353, *p* = 0.036; Fig. 2E). When we looked at the PSD length of non-perforated synapses we found that there was no difference in larger spines > 0.4 μm (F_(2, 1321)_ = 0.840, *p* = 0.432) but did see significant differences in smaller spines with FLASH treated mice having smaller PSDs than control and CONV treated mice (F_(2, 1158)_ = 5.190, *p* = 0.006, Control vs. FLASH: *p* = 0.019, CONV vs. FLASH: *p* = 0.01, Bonferroni’s multiple comparison tests; Fig. 2C). We also saw significant differences in HD. In synapses < 0.4 μm both CONV and FLASH treated mice had smaller HD than controls (F_(2, 1193)_ = 94.28, *p* < 0.0001, control vs. CONV: *p* < 0.0001, Control vs. FLASH: *p* < 0.0001, Bonferroni’s multiple comparison tests; Fig. 2F). For HD > 0.4 μm, irradiate mice had smaller HD (F_(2, 1344)_ = 4.964, *p* = 0.007, control vs. CONV: *p* = 0.006, Control vs. FLASH: *p* = 0.797, Bonferroni’s multiple comparison tests; Fig. 2F).

### FLASH and CONV mice have thinner myelin sheaths than controls

**Fig. 3:**
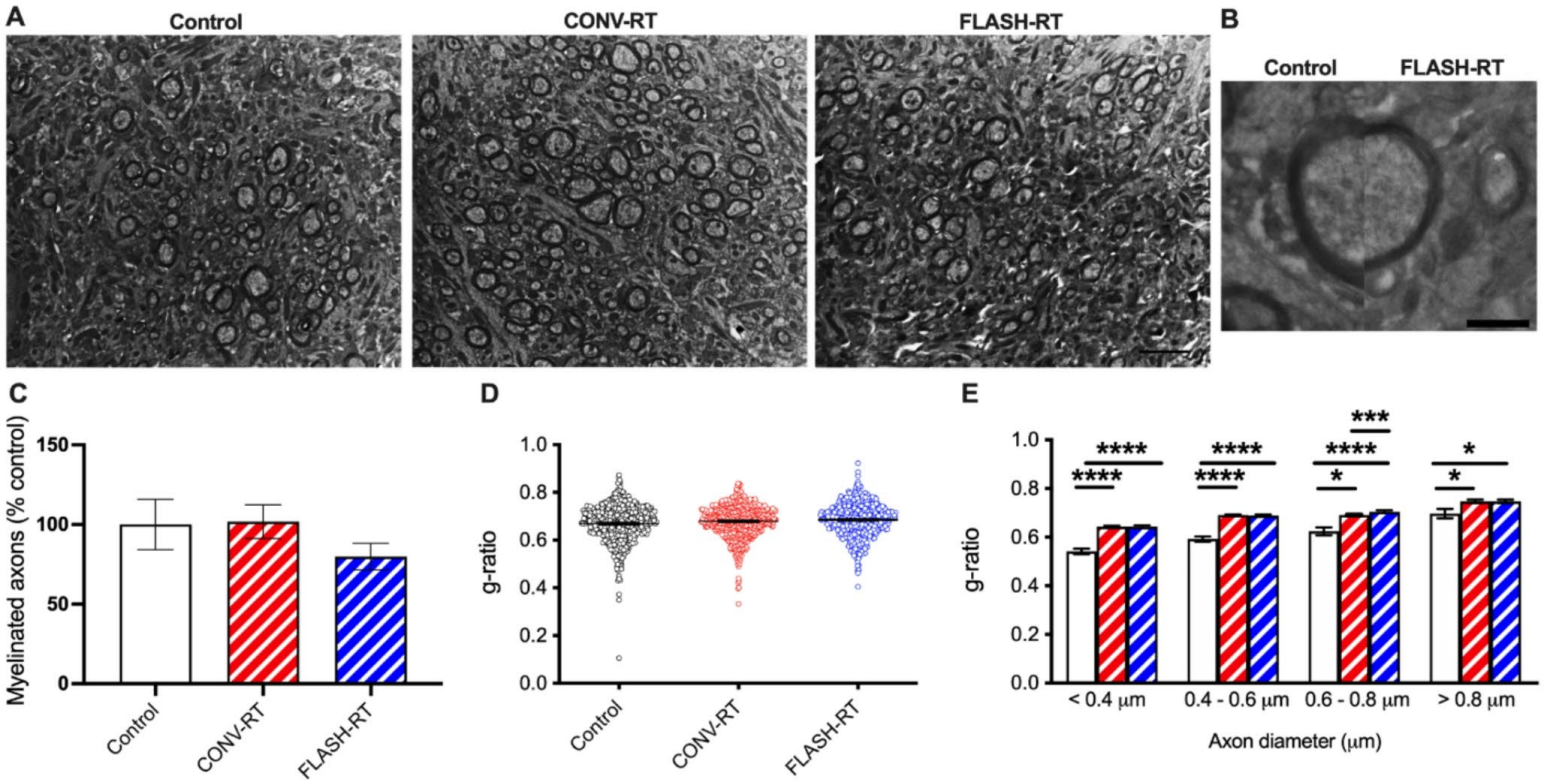
Radiation treatment results in a decrease in axon myelin sheath thickness. (**A**) Representative myelin images from all treatment groups. Scale bar = 500 nm. (**B**) Electron micrograph depicting a single axon of similar diameter in control mice (left) and FLASH mice (right). Scale bar 50 nm. (**C**) There was no significant difference in the percentage of myelinated axons between CONV, FLASH and control groups. Data represent group means ± SEM. Overall g-ratios are larger in radiation treated mice compared to controls (**D**) and when binned according to axon diameter (**E**). Data represents individual measurements ± SEM. *p < 0.05, **p < 0.01, ***p < 0.001, ****p < 0.0001

We analyzed the morphology of axons in all three groups of mice (Table 1). Representative images of myelinated axons are depicted in Fig. 3A and B. We found that CONV treatment restored the percent of myelinated axons to levels similar to controls while FLASH treated animals had fewer myelinated axons, however these changes did not reach significance (F_(2, 12)_ = 3.518, *p* = 0.079, one-way ANOVA; Table 1; Fig. 3C). We then measured the mean g-ratio (the ratio between the diameter of an axon and the diameter of the fiber including myelin) for all axons (Fig. 3D). We did observe that the myelin sheath of irradiated mice was thinner than control as seen by a larger g-ratio value. In particular, the g-ratio of FLASH irradiated fibers was significantly increased when compared with controls indicating a thinner myelin sheath (F_(2, 1637)_ = 6.477, *p* = 0.002, one-way ANOVA; control vs. CONV: *p* = 0.051, Control vs. FLASH: *p* = 0.001, Tukey’s multiple comparison tests, Fig. 3E). When we analyzed g-ratio within classes of fibers binned for their axonal diameter, decreased myelin thickness was present for all diameter sizes for both CONV and FLASH irradiated animals compared to controls (diameter < 0.4 mm: F_(2, 502)_ = 55.61, *p* = 0.0001, one-way ANOVA, control vs. CONV: *p* < 0.0001, Control vs. FLASH: *p* < 0.0001, Tukey’s multiple comparison tests; diameter 0.4–0.6 mm: F_(2, 706)_ = 70.57, *p* < 0.0001, one-way ANOVA, control vs. CONV: *p* < 0.0001, Control vs. FLASH: *p* < 0.0001, Tukey’s multiple comparison tests; diameter 0.6–0.8 mm: F_(2, 1039)_ = 17.12, *p* < 0.0001, one-way ANOVA, control vs. CONV: *p* = 0.026, Control vs. FLASH: *p* < 0.0001, CONV vs. FLASH: *p* = 0.0002, Tukey’s multiple comparison tests; diameter > 0.8 mm: F_(2, 105)_ = 4.998, *p* = 0.008, one-way ANOVA, control vs. CONV: *p* = 0.02, Control vs. FLASH: *p* < 0.02, Tukey’s multiple comparison tests; Fig. 3E).

### Dendritic complexity, spine density and spine type are not affected by radiation dose or dose-rate

Additional studies were performed using Thy1-eGFP-expressing mice exposed to CONV and FLASH RT, to determine whether certain changes in neuronal morphology could be found. Data revealed that the structure of several morphological parameters of CA1 neurons were relatively insensitive to changes in dose rate (Supplemental Fig. 3, Table 2). Additional analyses of spine class types were performed that also did not reveal significant dose or dose rate dependent changes (Supplemental Fig. 4, Table 3).

**Table 2:** Summary of the numbers and quantitative morphological data of neurons and synapses for each treatment group derived from EGFP-labeled brain sections

	Control	CONV	FLASH
	(Mean ± SEM)	(Mean ± SEM)	(Mean ± SEM)
Spine Head Diameter	0.51 ± 0.021	0.6 ± 0.02	0.54 ± 0.02
Dendritic Branches	4.43 ± 0.39	3.46 ± 0.32	3.93 ± 0.43
Dendritic Area	1135.17 ± 139.60	1020.27 ± 115.96	1075.17 ± 108.32
Spine Volume	40.83 ± 7.20	56.84 ± 17.44	62.62 ± 18.83
Filament Volume	202.55 ± 27.00	250.20 ± 33.48	245.20 ± 28.65
Filament Length	453.71 ± 47.63	365.35 ± 35.92	409.50 ± 40.08
Spine Count	242.17 ± 32.32	205.85 ± 26.98	216.20 ± 23.93

**Table 3:** Summary of spine classifications along CA1 dendritic segments for each treatment group derived from EGFP-labeled brain sections

	Stubby	Mushroom	Long/Thin/Filopodia	Average Spine Count
	(Mean ± SEM)	(Mean ± SEM)	(Mean ± SEM)	(Mean ± SEM)
Control	6.22 ± 1.28	11.11 ± 1.470	40.11 ± 8.16	61.89 ± 8.23
CONV	6.78 ± 2.48	8.44 ± 2.163	55.33 ± 9.95	70.56 ± 10.55
FLASH	5.78 ± 2.51	3.56 ± 0.556	58.67 ± 2.59	69.65 ± 5.44

### Excitatory/inhibitory synapse density is not altered by radiation dose or dose-rate

**Fig. 4:**
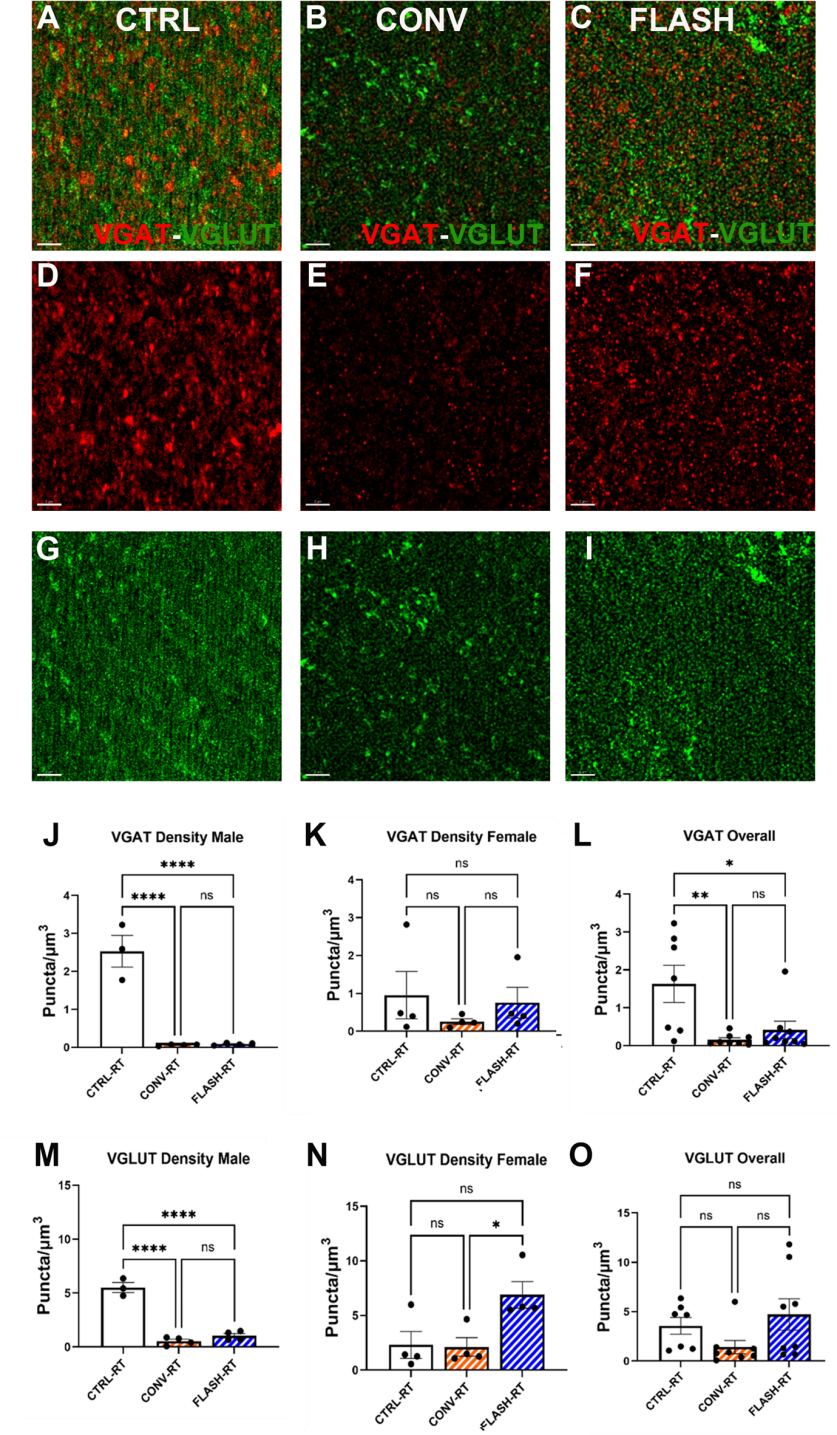
Super resolution microscopy analysis of excitatory and inhibitory synaptic vesicles (VGLUT, VGAT) post-CONV and FLASH dose-rate irradiations. **(A-C)** Representative full view z stacks of colocalized VGAT (Red) and VGLUT (Green) immunostaining within the CA1 *stratum radiatum* of C57Bl/6 male mice exposed to 0 Gy (CTRL RT), or 3 × 10 Gy CONV or FLASH irradiation, **(D-F)** VGAT (Red) alone, and **(G-I)** VGLUT (Green) alone (Scale bar 2 μm). 3D algorithm-based volumetric quantification of VGAT^+^**(J-L)** and VGLUT^+^**(M-O)** immunoreactive puncta. Data represent mean ± SEM (*N* = 4 mice/group/sex J, K,M, N; 8/grp sexes combined L, O). One-way ANOVA followed by Bonferroni’s multiple comparison. *p < 0.05, **p < 0.01, ****p < 0.0001

To determine the impact of cranial irradiation and dose-rates on inhibitory and excitatory vesicular trafficking markers, immunofluorescence staining, super resolution microscopy, and 3D algorithm-based volumetric quantification of immunoreactive puncta for excitatory vesicular glutamate transporter 1 (VGLUT) and inhibitory vesicular GABA transporter (VGAT) was conducted within the CA1 *stratum radiatum* of male and female C57Bl/6 mice exposed to cranial irradiation (Fig. 4). We found a significant decline in number of VGAT and VGLUT immunoreactive puncta following 3 × 10 Gy in either CONV (*p* < 0.0001) or FLASH (*p* < 0.0001) irradiation compared to unirradiated controls in male animals (Fig. 4A-F, J, M). However, we did not find significant differences between CONV and FLASH dose-rates for the number of VGAT and VGLUT puncta in those male mice (Fig. 4J, M). For female mice a trend for a decrease in VGAT immunoreactive puncta was observed for the mice exposed to 3 × 10 Gy of CONV dose-rate irradiation, but it did not reach statistical significance (Fig. 4K). Conversely in these same female mice, an increase in VGLUT puncta was observed in the 3 × 10 Gy FLASH irradiated females that was different from both control and 3 × 10 Gy CONV females (*p* < 0.05; Fig. 4N). When these data for both sexes were combined, significant decreases in overall VGAT levels were observed in mice exposed to either 3 × 10 Gy of either CONV (*p* < 0.001) or FLASH (*p* < 0.05) irradiation compared to unirradiated controls (Fig. 4L) while no overall changes in levels of VGLUT were observed (Fig. 4O).

## Discussion

In the present study, we used ultrastructural analyses of neuronal populations by EM and analysis of fluorescently labeled neurons in the CA1 by confocal microscopy to investigate the impact of radiation exposure and dose rate modulation on mature and arbored subsets of neurons located in the pyramidal layer of the CA1 and prelimbic/infralimbic region of the medial prefrontal cortex (PFC). We found that these pyramidal neurons are radiation resistant and dose rate insensitive, while analyses of excitatory vesicular glutamate transporter 1 and inhibitory vesicular GABA transporter (i.e. glutamatergic/GABAergic VGLUT/VGAT) puncta in the CA1 revealed dose-dependent reductions in synapse density, they were not found to depend on dose-rate.

The mechanisms underlying radiation-induced cognitive dysfunction are complex and multifactorial, involving multiple cellular subtypes that directly and indirectly regulate neurotransmission. As direct mediators of this process, neurons are central candidates, and the temporal coincidence between radiation-induced cognitive decrements and changes in neuronal structure provides one plausible explanation. A logical extension of this tenet would presume that different neurons across various regions of the brain would exhibit different sensitivities to such change, and our data here support that idea, where CA1 and PFC pyramidal cell neurons were more resistant to radiation-induced reductions in dendritic morphology compared to granule cell neurons in the hippocampal dentate detailed in a prior study (Montay-Gruel et al. 2019). The foregoing was corroborated using two independent techniques for quantifying morphologic parameters, where dendritic complexity and spine density of dye-loaded and intrinsically fluorescent pyramidal cell neurons were also relatively unaffected by changes in dose-rate. While changes in non-perforated synapses were found in the irradiated cohorts analyzed by EM, ascribing how such alterations might impact the wide range of behavioral tasks analyzed in our past CONV and FLASH radiation studies remains uncertain. Data do indicate however that radiation exposure, especially CONV, had a larger impact on non-perforated synapses. While dose rate dependent differences were small, findings suggest that FLASH was able to preserve perforated synapses. Numerous studies have found that increased levels and/or size of perforated synapses enhance synaptic strength (Geinisman et al. 1993; Nicholson et al. 2004; Toni et al. 2001), suggesting that FLASH maintains neurotransmission by preserving perforated synapses. This idea does corroborate our past findings showing a preservation of long-term potentiation in the hippocampus and PFC (Alaghband et al. 2023a; Allen et al. 2022; Limoli et al. 2023). Sex-dependent changes in the density of VGLUT puncta in female mice versus male mice exposed to FLASH were found. Reasons why FLASH might preferentially upregulate this excitatory protein in female mice are not clear. Radiation exposure was found to reduce the VGLUT/VGAT synapse density, and while trends pointed to preservation of this loss after FLASH-RT, significance was not found across the cohorts analyzed.

Not all reports in the literature have found FLASH sparing, where conflicting results have been reported for GI toxicities using proton FLASH (Velalopoulou et al. 2021; Bell et al. 2024) and electron FLASH (Venkatesulu et al. 2019), while the latter was likely performed at suboptimal dose rates. FLASH has also been shown to induce radionecrosis in cat patients, where dose limiting toxicities were exceeded by a by a single isodose of 30 Gy (Rohrer Bley et al. 2022). In the brain, a single FLASH dose of 14 Gy was not found to provide neurocognitive sparing (Montay-Gruel et al. 2021), while a single dose of 10 Gy (FLASH or CONV) was found to elevate the expression levels of C1q and C3 compared to the controls, key components of the proinflammatory complement cascade (Montay-Gruel et al. 2020).

While this investigation sought to uncover whether distinct neuronal populations exhibited differences in radiation-induced structural plasticity, it also sought to determine whether such changes were dose-rate dependent. Here, the radioresistance of pyramidal cell structural plasticity precluded the FLASH sparing of the morphologic determinates evaluated in the CA1 and mPFC. Past work implementing time-lapsed 2-photon microendoscopy in the CA1 of live mice has shown the temporal dynamics of dendritic spine turnover to differ across brain regions (Berry and Nedivi 2017), suggesting that the transience of hippocampal-dependent memory is linked to the turnover of hippocampal synapses (Attardo et al. 2015), reviewed in (Berry and Nedivi 2017). Several studies focused on aging and neurodegeneration have linked changes in dendritic spine morphology to functional impairments and cognition (Bloss et al. 2010, 2011, 2013); Price et al. 2014 #3180; Steele et al. 2014 #2620]. Past data implementing the objects in updated locations task, shows routinely that the update session reliant on hippocampal memory exhibits CONV radiation-induced deficits in both sexes, while various versions of the recall memory tasks show differential responses between the sexes known to be reliant on the PFC, dose rate and dose fractionation (Alaghband et al. 2023a; Allen et al. 2022). Differences in the susceptibility of different neuronal populations to radiation dosing and dose rate might provide a plausible explanation for these differential cognitive effects. Irradiation is likely to alter basal turnover rates of many critical synaptic elements across the entire brain and given the protracted nature of such changes it is hard to dismiss the relationship between neuronal morphology and cognition. Clearly many cellular mechanisms converge to impact cognition, and the benefits of FLASH-RT cannot be solely linked to the integrity of the dendritic tree, synapse density or morphology across different neuronal populations. While the unique memory sparing capabilities of FLASH radiotherapy have provided a potentially new avenue for resolving quality of life concerns in brain tumor survivors, such benefits likely extend beyond neuronal structure and across multiple cell types in the brain.

## Electronic supplementary material

Below is the link to the electronic supplementary material.

Supplementary Material 1

## Data Availability

No datasets were generated or analysed during the current study.

## Declarations

### Disclaimer

The opinions expressed herein are those of the authors and are not necessarily representative of those of the government of the United States, the Uniformed Services University of the Health Sciences, the Department of Defense (DoD), or the United States Army, Navy or Air Force or the Henry M. Jackson Foundation for the Advancement of Military Medicine Inc.
