# Supplementary material for: Structural plasticity of pyramidal cell neurons measured after FLASH and conventional dose-rate irradiation
Source: Brain Struct Funct. 2025 Mar 1;230(2):41. doi: 10.1007/s00429-025-02902-y (PMC11872753; doi:10.1007/s00429-025-02902-y)
Supplement: Supplementary file 1 — Supplementary Material 1 [file 429_2025_2902_MOESM1_ESM.docx]

**Supplemental Results**


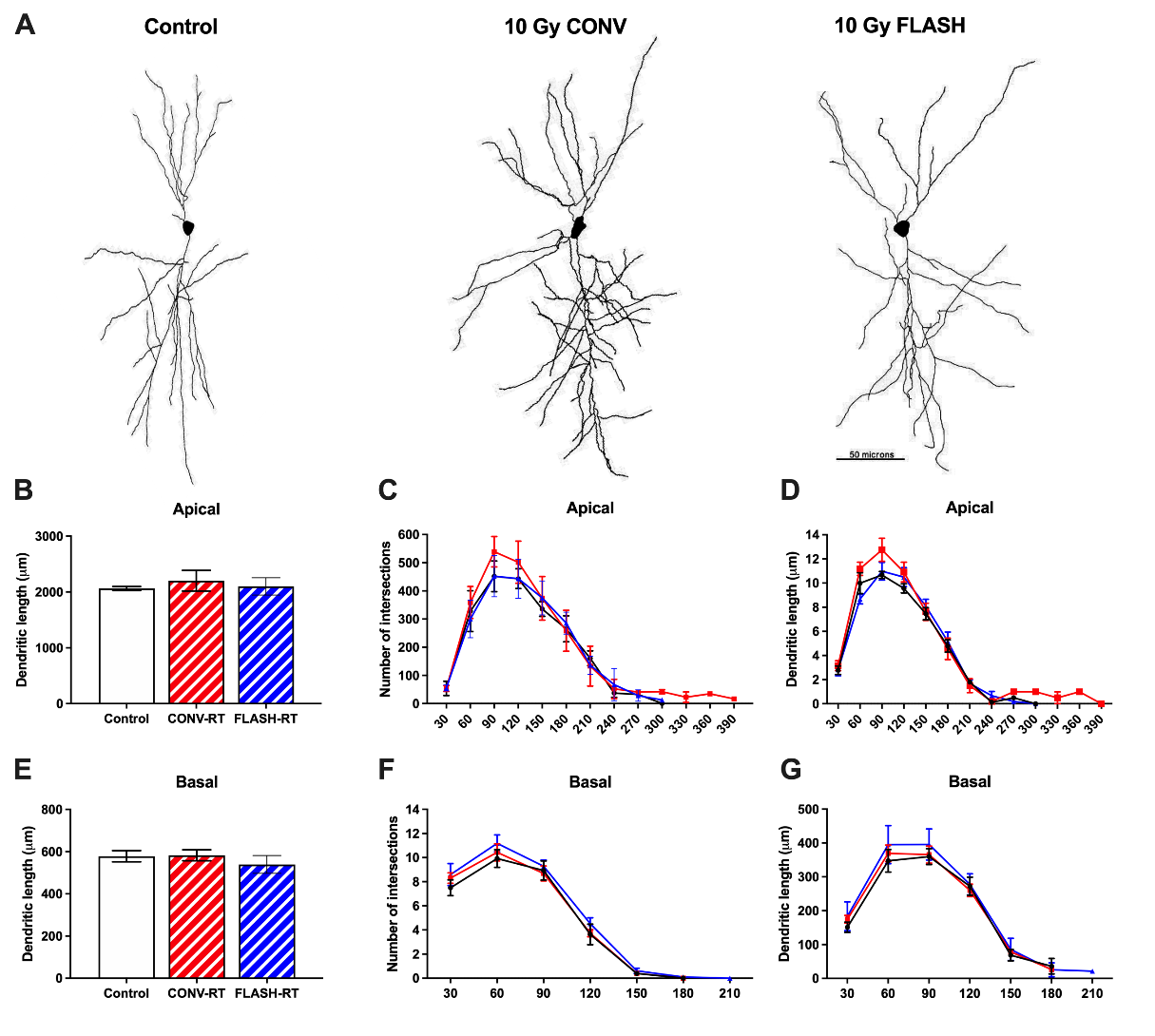
***FLASH and CONV treatment had no effect on neuronal complexity.*** A total of 14 animals and a minimum of 5 neurons per animal met the inclusion criteria for use in neuronal reconstructions (**Table 1**). Representative examples of CA1 dendritic arbor reconstructions of FLASH, CONV and control neurons are depicted in **Suppl. Fig. 1A.** We found no significant difference in apical or basal dendritic length between all three treatment groups (apical: F_(2, 11)_ = 0.22; *p* = 0.81 and basal: F_(2,11)_ = 0.53, *p* = 0.60, one-way ANOVA; **Suppl. Fig. 1B, E**). We then performed a Sholl analysis to identify any further changes in morphological complexity that may occur in response to different radiation treatments. When comparing the numbers of intersections and the amount of dendritic length at specific distances from the soma on both apical and basal dendrites, we found no significant main effect of treatment (**Suppl. Fig. 1 C, D and F, G respectively**).

**Supplemental Figure 1. Cranial irradiation with either CONV or FLASH irradiation had no effect on CA1 pyramidal neuronal complexity.** (**A**) Representative cell tracings from each treatment group. Scale bar 50 µm. (**B**) Analysis of apical dendritic length. (**C,D**) Sholl analyses of apical dendrites. (**E**) Analysis of basal dendritic length. (**F,G**) Sholl analyses of basal dendrites. Data represent mean ± SEM.


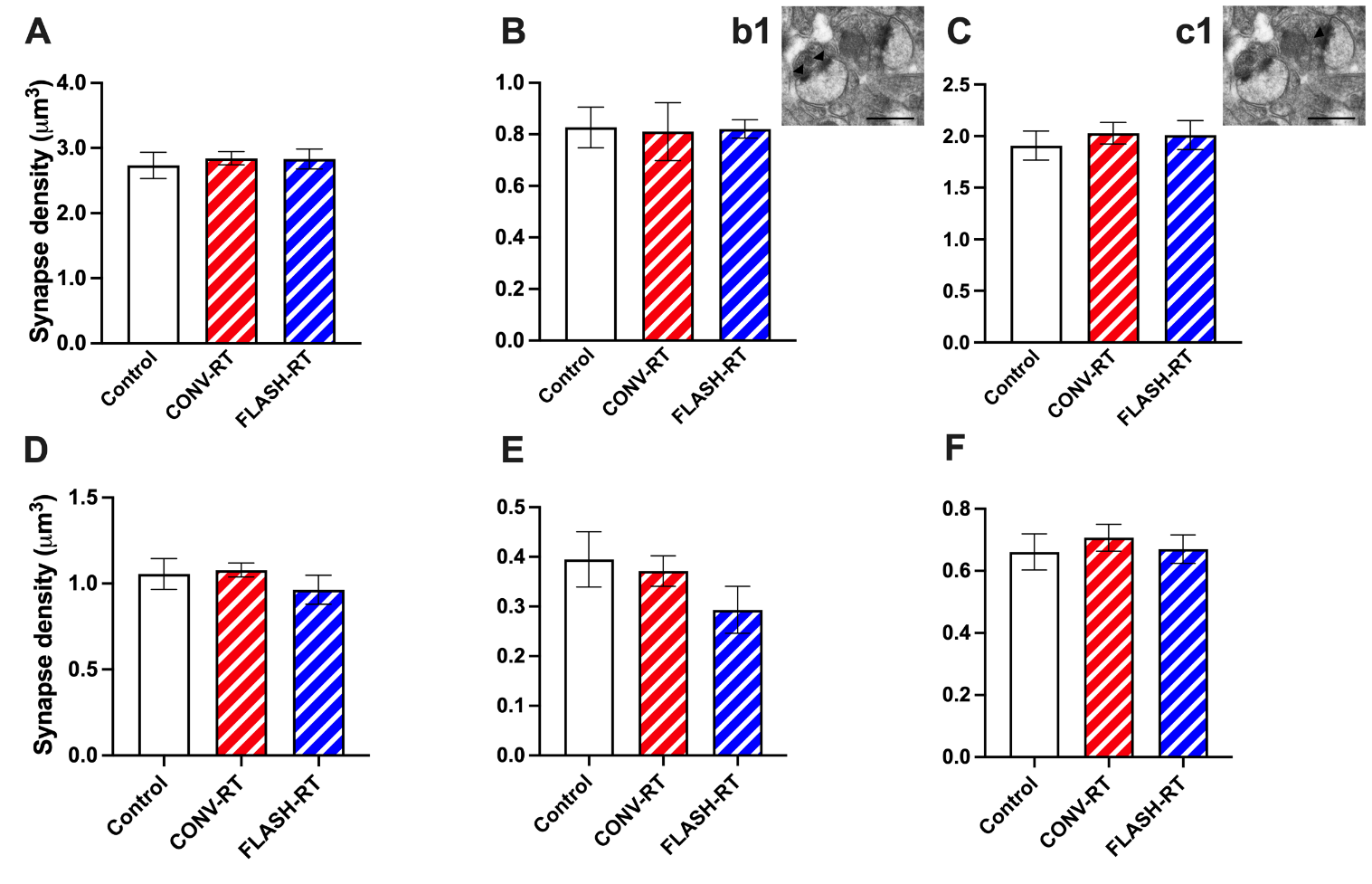
***FLASH and CONV treatment had no effect on synapse density in the CA1 and mPFC.*** Analysis of total synapse density as well as perforated and non-perforated synapse density in hippocampal CA1 pyramidal neurons revealed no significant differences between control, CONV and FLASH irradiated mice (2.735 ± 0.202 synapses/µm^3^), CONV (2.839 ± 0.101 synapses/µm^3^) and FLASH mice (2.831 ± 0.153 synapses/µm^3^ **(Suppl.** **Fig. 2 A-C**). The density of different types of synapses, perforated versus non-perforated, was assessed further. Neither CONV nor FLASH treatments changed the density of perforated synapses compared to control mice (0.413 ± 0.039), CONV (0.405 ± 0.056) and FLASH mice (0.410 ± 0.018) perforated synapses/µm^3^ or non-perforated synapses compared to control mice 2.321 ± 0.169, CONV 2.434 ± 0.086, FLASH 2.420 ± 0.146 non-perforated synapses/µm^3^) **(Suppl.** **Fig. 2 B, C**).

**Supplemental Figure 2. Cranial irradiation did not impact CA1 or mPFC synapse density of mice exposed to either CONV or FLASH irradiation.** There is no significant difference in (**A,D**) total synapse density, (**B,E**) perforated and (**C,F**) nonperforated synapse density in both CA1 hippocampal neurons (top row) and mPFC (bottom row). Inset images depict perforated synapses (arrowheads in **b1**) and nonperforated synapses (arrowhead in **c1**). Scale bar in b1 and c1 = 50 μm.

We also examined the axospinous synapse density in the prelimbic/infralimbic region of the mPFC (**Suppl. Fig. 2 D-F**). We found no significant differences in total synapse density between control mice, CONV mice and FLASH mice (**Suppl. Fig. 2 D).** The density of different types of synapses, perforated versus non-perforated, was assessed further. While CONV mice seemed to have a similar perforated synapse density as controls, FLASH-treated mice had fewer perforated synapses, this difference did not reach significance (F_(2, 12)_ = 1.358, *p* = 0.294, one-way ANOVA; **Suppl. Fig 2E**). There was no significant difference in non-perforated synapse density among groups (F_(2, 12)_ = 0.2372, *p* = 0.7924, one-way ANOVA; **Suppl.** **Fig 2F**).

***Dendritic complexity and spine density is not affected by radiation dose or dose-rate.***

Using Thy1-eGFP-expressing mice exposed to CONV and FLASH RT, we analyze CA1 pyramidal neuronal spine density, number of dendritic branches and dendritic (filament) volume within the apical dendrites in the SR (**Suppl. Fig. 3**). This analysis was facilitated by Imaris (v10) filament reconstruction module as described previously (Parihar et al. 2015). We did not find significant differences in spine density per 100 μm of the dendritic section following either CONV or FLASH- dose-rate irradiations (**Suppl. Fig 3 G-I**). Similarly, we did not find statistical significance between unirradiated controls, CONV- or FLASH-RT for dendritic parameters including dendritic branch numbers and dendritic volume.


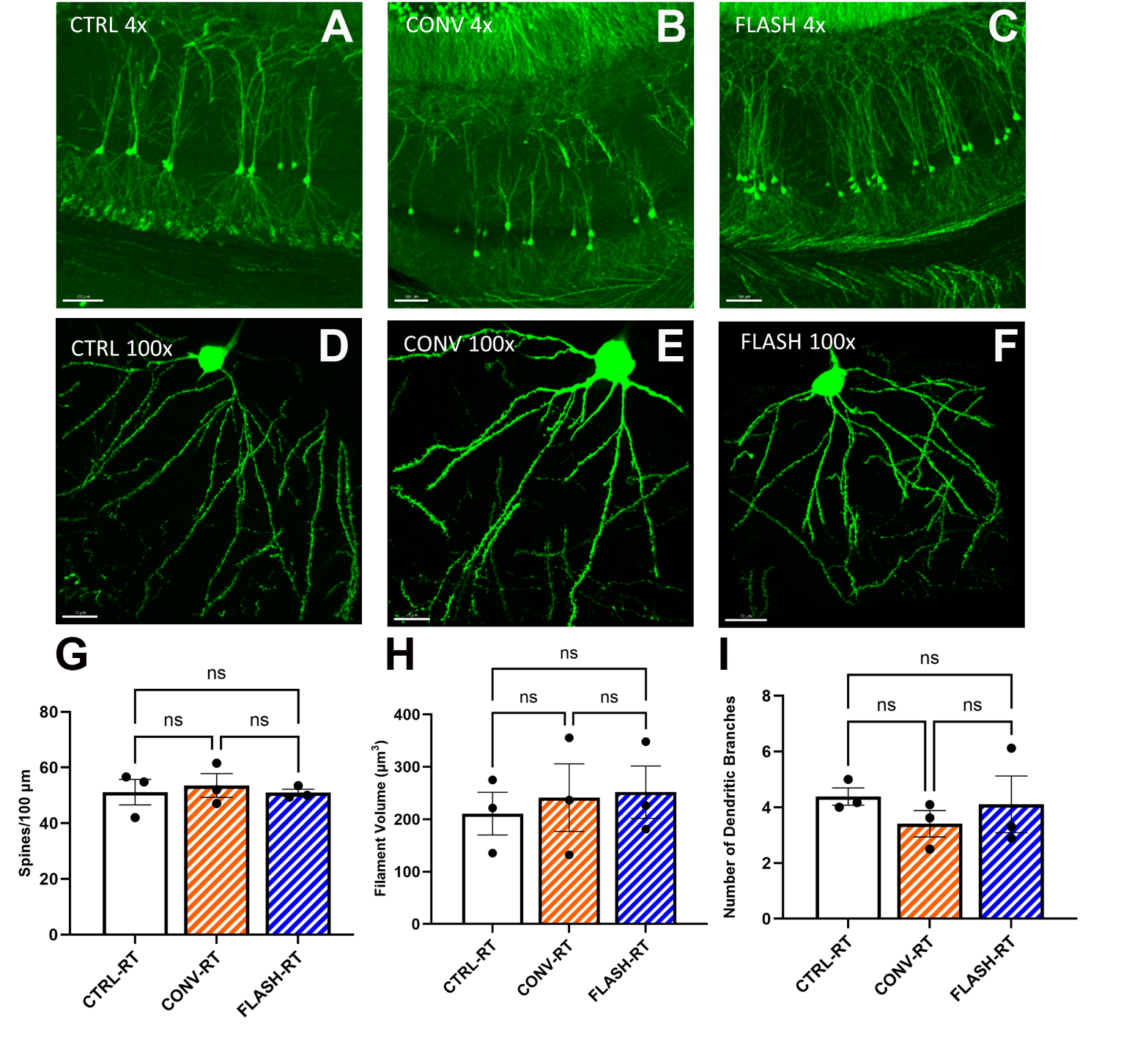


**Supplemental Figure 3.** Cranial irradiation did not impact CA1 pyramidal neuron morphology and spine density in the Thy1-eGFP transgenic brains of mice exposed to either CONV or FLASH irradiation **(A-C).** Representative full view z stacks of CA1 pyramidal neurons showing eGFP+-fluorescent apical dendrites emanating through the CA1 *stratum radiatum*. Scale bars 400 μm and 15 μm, respectively **(D-F).** 3D algorithm-based filament and volumetric quantification of apical dendrite spine density, number of dendritic branches and filament volumes showed no significant differences between Control (CTRL), CONV and FLASH RT **(G-I).** Data represent mean ± SEM (N=3 mice/group). One-way ANOVA.


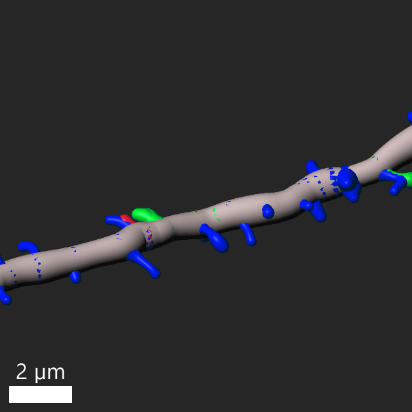

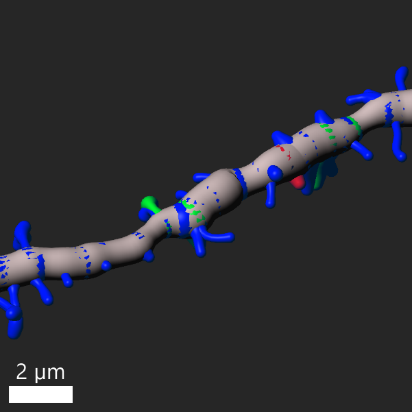

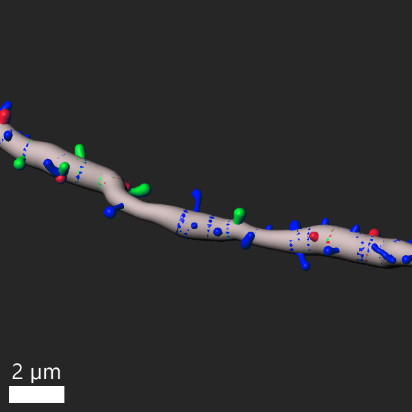


C

B

A


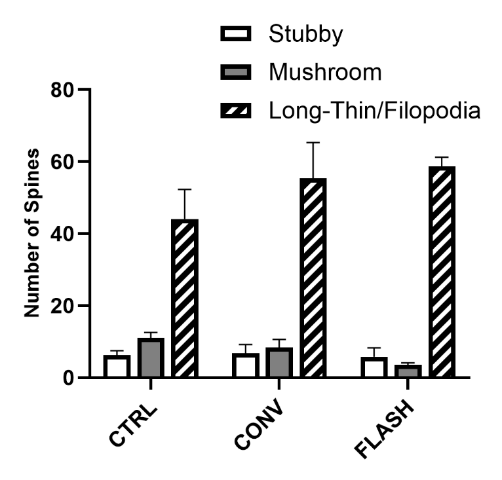

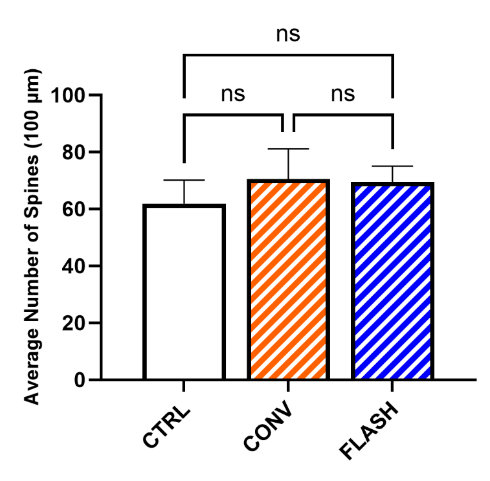


D

E

**Supplemental Figure 4.** Cranial irradiation or dose rate did not alter the morphologically distinct spine. **(A-C).** Representative reconstruction of dendritic filament (grey) showing different spine types, including filopodia (long-thin, blue), mushroom (green), and stubby (red) spines in the Control (**A**), CONV (**B**), and FLASH (**C**) irradiated brains. **(D-E)** Quantification of reconstructed apical dendritic segments from the CA1 pyramidal neurons (10 µm of dendritic segments) show that either exposure to CONV or FLASH irradiation does not have a significant impact on filopodia (long-thin), mushroom, or stubby spine types. Data are presented as Mean ± SEM (n = 3 mice/group). Scale bar, 2 µm, **A-C.**

**Supplemental Reference**

Parihar VK, Pasha J, Tran KK, Craver BM, Acharya MM, Limoli CL (2015) Persistent changes in neuronal structure and synaptic plasticity caused by proton irradiation. Brain structure & function 220 (2):1161-1171. doi:10.1007/s00429-014-0709-9
